# Establishment and preliminary application of personalized three‐dimensional reconstruction of thyroid gland with automatic detection of thyroid nodules based on ultrasound videos

**DOI:** 10.1002/acm2.14332

**Published:** 2024-03-25

**Authors:** Huang Yandong, Zhang Shiqi, Jia Lanting, Hu Wenxin, Chen Leyao, Huang Hejing

**Affiliations:** ^1^ Department of Ultrasound Second Affiliated Hospital of Naval Medical University Shanghai China; ^2^ School of Data Science and Engineering East China Normal University Shanghai China

**Keywords:** 3D reconstruction, location, nodule, thyroid, ultrasound

## Abstract

**Purpose:**

A well display of the spatial location of thyroid nodules in the thyroid is important for surgical path planning and surgeon‐patient communication. The aim of this study was to establish a three‐dimensional (3D) reconstruction method of the thyroid gland, thyroid nodule, and carotid artery with automatic detection based on two‐dimensional (2D) ultrasound videos, and to evaluate its clinical value.

**Methods:**

Ultrasound videos, including the thyroid gland with nodule, isthmus of thyroid gland, and ipsilateral carotid artery, were recorded. BC‐UNet, MTN‐Net, and RDPA‐U‐Net network models were innovatively employed for segmentation of the thyroid glands, the thyroid nodules, and the carotid artery respectively. Marching Cubes algorithm was used for reconstruction, while Laplacian smoothing algorithm was employed to smooth the 3D model surface. Using this model, 20 patients and 15 surgeons completed surveys on the effectiveness of this model for the pre‐surgery demonstration of nodule location as well as surgeon‐patient communication.

**Results:**

The thyroid gland with nodule, isthmus of gland, and carotid artery were reconstructed and displayed. With the 3D model, the understanding of the spatial location of thyroid nodules improved in all three surgeon groups, eliminating the influence of professional levels. In the patient survey, the patients’ understanding of the thyroid nodule location and procedure for surgery were significantly improved. In addition, with the 3D model, the time for doctors to explain to patients was significantly reduced (16.75  vs. 8.85 min, *p* = 0.001).

**Conclusion:**

To our knowledge, this is the first report of constructing a 3D thyroid model using a deep learning technique for personalized thyroid segmentation based on 2D ultrasound videos. The preliminary clinical application showed that it was conducive to the comprehension of the location of thyroid nodules for surgeons and patients, with significant improvement on the efficiency of surgeon‐patient communication.

## 1 INTRODUCTION

The prevalence of thyroid nodules among the general population has been estimated as upwards of 67%.^1^ Surgery is the main treatment option for thyroid cancer and some benign nodules.^2^, ^3^ In most cases, thyroid ultrasound examination is the most important imaging method preoperatively. It is characterized by wide availability, rapidity, cost efficiency, and radiation‐free. The shape, size, and location of the thyroid nodule as well as its relationship with thyroid capsule could be clearly displayed by ultrasound. For the surgeons, a clear understanding of the spatial location of the thyroid nodules is essential for surgical path planning and tumor‐free resection. In addition, the information concerning the nodules mentioned above should be thoroughly explained to the patient by the surgeon preoperatively, since the patient's preferences play a role in determining the surgical procedure.^4^

However, conventional thyroid ultrasound could not fully meet the clinical needs for the following reasons: (1) The clinically applied ultrasound images are two‐dimensional. Although the location of the nodule could be displayed in the cross‐section and longitudinal section on ultrasound, it could not provide the spatial relationship of the thyroid nodule with the whole gland, lacking global anatomical view. (2) Generally, the surgeons are provided with static ultrasound images and ultrasound report of the patients with thyroid nodules via PACS (Picture Archiving and Communication Systems) system. It is challenging for surgeons to understand ultrasound images and comprehend the spatial location of the thyroid nodule. Moreover, the description of nodule location in the ultrasound report is ambiguous (generally use the term “upper,” “middle,” or “lower” poles of the thyroid) and sometimes it is completely different from the preoperative assumption of the surgeon. (3) Most patients do not have enough medical knowledge. It is difficult for surgeons to communicate with patients and explain the disease by ultrasound images before the surgery.

To solve the above problems, the investigators conducted several studies. Some investigators developed preoperative ultrasound‐guided drawing by hand for locating thyroid nodules based on ultrasound images, which proved useful for locating and searching thyroid micronodules during surgery,^5^ while the disadvantage of this method was that it was time‐consuming, inaccurate, and could not provide spatial information. In 2019, Dayeong et al. attempted to use a three‐dimensional (3D)‐printed thyroid cancer phantom for surgeon‐patient communication.^4^ However, this phantom was produced from segmented head and neck contrast‐enhanced computed tomography (CT) from a patient with thyroid nodules. The cost of producing the phantom and additional radiation from CT had to be weighed, since CT was not a routine procedure for thyroid surgery. In 2021, Seok et al. used a U‐Net‐based deep learning architecture and a mesh‐type 3D modeling technique to fabricate the personalized 3D model based on data from CT.^6^ However, this method was still based on CT images and additional radiation was unavoidable.

Thus, in our study, we aim to establish a three‐dimensional (3D) reconstruction method of the thyroid gland, thyroid nodule and carotid artery with automatic detection based on two‐dimensional (2D) ultrasound videos, which could clearly display the anatomical location of the thyroid nodule and could be viewed from all angels. It is easy to understand, radiation‐free, and cost‐effective. The 3D model is conducive to the comprehension of the location of thyroid nodules for surgeons and patients, with significant improvement on the efficiency of surgeon‐patient communication.

## 2 METHODS

### 2.1 Ultrasound video acquisition

This study was approved by the institutional review board, and informed consent was obtained from all patients and surgeons. The patients participated received conventional B‐mode ultrasound examination of the thyroid gland using a 14 MHz linear array probe of resona7 ultrasound system (Mindray Bio‐Medical Electronics, Shenzhen, China). All patients were positioned in supine with dorsal flexion of the head. A continuous transverse ultrasound video recording from the appearance of the upper thyroid pole to the disappearance of the lower thyroid pole were collected by the same experienced examiner. The thyroid lobe with nodule, isthmus of thyroid gland, and ipsilateral carotid artery were included in the ultrasound video.

### 2.2 The 3D reconstruction of thyroid gland, nodules, and carotid artery

First, we designed a BC‐UNet to segment the thyroid gland in ultrasound video, which consisted of a temporal encoder and a spatial encoder. The spatial encoder was used to segment the thyroid gland in each frame of the ultrasound image. It improved the UNet^7^ by embedding the ASPP^8^ module to enable the network to acquire multi‐scale features of the thyroid gland, thus enabling the network to recognize different sizes of thyroid sections. The temporal encoder, on the other hand, consisted of the BiConvLSTM,^9^ which could combine the contextual information of the video to optimize the result of the current frame from both the before and after temporal directions, thus enabling the network to achieve accurate segmentation of the thyroid gland (Figure S1).

Second, we designed the MTN‐Net^10^ based on multi‐scale feature fusion for detecting and segmenting thyroid nodules in ultrasound images. The network used the Trident Network^11^ as the baseline, where the Trident Block^11^ was used to fully capture the multi‐scale features of thyroid nodules so that the network could identify thyroid nodules of different sizes. We also proposed a TN‐NMS^10^ algorithm for combining the results of multiple branches, which could effectively suppress the false detection of internal nodules. Finally, a semantic segmentation branch was embedded in the detection network, which could accurately segment thyroid nodules based on the detection results (Figure S2).

Third, the carotid artery was segmented by RDPA‐U‐Net network model, which was improved based on U‐Net. The convolutional layer in encoder and decoder structure was replaced by ring residual module to solve the network degradation problem caused by too deep network. At the same time, a dual‐path attention module integrating spatial attention module and pyramid squeeze attention module in parallel was used to improve the skip connection part of U‐Net. In this way, multi‐scale spatial information was fused to enrich the feature space, so as to obtain the accurate segmentation result of carotid artery (Figure S3).

Fourth, based on the segmentation results of the thyroid gland, nodules, and carotid artery, we used the Marching Cubes^12^ algorithm to construct 3D models of the thyroid gland, nodules, and carotid artery, and employed the Laplacian smoothing algorithm to smooth the surface of the generated 3D models, so that the generated 3D models could more accurately represent the real shape, size and location of the thyroid gland, nodules and carotid artery.

Finally, all proposed networks mentioned above were implemented in PyTorch 1.8.1, and were trained on two NVIDIA Tesla P100 GPUs with a batch size of 16. Moreover, the models were trained with the stochastic gradient descent optimizer and the learning rate of warmup and cosine annealing for 50 epochs, whose learning rate increased linearly to 0.05 in the first 1000 iterations, then decreased gradually in the form of cosine annealing. The total training time for the models was 20 h, and the inference time for each image was 0.85 s. The source code of the thyroid tool is available on Github (https://github.com/shcz‐ecnu/thyroid‐tool). For more detailed information, please contact the corresponding author.

### 2.3 The survey

Two surveys were conducted. The first survey aimed to test whether the 3D model could help surgeons understand the location of thyroid nodules. One patient with a thyroid nodule was prepared for the survey. The thyroid ultrasound report, the conventional ultrasonic images of the thyroid nodule, and the corresponding 3D reconstruction model were available. Five surgical residents, five attending surgeons, and five associate chief surgeons participated in the study. First, all participants were provided with the patient's ultrasonic images and report. After that, all participants were provided with the patient's 3D reconstruction model. Finally, all participants completed a questionnaire to evaluate whether the use of 3D models would be helpful in understanding the anatomical location of thyroid nodule. Scores were recorded as 1 = don't understand at all, 2 = don't understand, 3 = not clear, 4 = understand, and 5 = fully understand.

The second survey aimed to test whether the 3D model could help patients understand the location of thyroid nodules and promote surgeon‐patient communication. Twenty patients who were scheduled to undergo unilateral thyroidectomy in our hospital from January to April 2023 were selected. The patients were randomly divided into experimental group and control group, with 10 people in each group. All patients underwent routine thyroid ultrasound examination and an ultrasound report was generated. In the experimental group, the 3D reconstruction model was produced for each patient based on ultrasound video of the unilateral thyroid lobe. One surgeon conducted conversations for all patients based on ultrasound report with (experimental group) or without (control group) 3D reconstruction models. A survey was conducted immediately after each conversation to quantitatively evaluate the value of 3D reconstruction technology in assisting patients to understand thyroid anatomy and surgical process. The contents of the questionnaire include: (1) the understanding of the thyroid nodule location and the procedure for thyroid surgery. Scores were recorded as 1 = don't understand at all, 2 = don't understand, 3 = not clear, 4 = understand, and 5 = fully understand; (2) the time consumption of surgeon‐patient communication for each patient.

### 2.4 Statistical analysis

The descriptions of demographic and clinical characteristics were documented as mean ± SD (standard deviation) for continuous variables, and frequencies (percentages) for categorical variables. Student's *t*‐test and Chi‐square test were applied to compare the difference of baseline characteristics between two groups for continuous and categorical variables, respectively. Multiple comparisons were performed by analysis of variance (ANOVA). One‐way ANOVA was followed by LSD test. If the homogeneity of variance is significant (*p* < 0.05), one‐way ANOVA was followed with Dunnett's T3 test. *p* value < 0.05 was regarded as statistically significant. SPSS (SPSS Inc., Chicago, USA; version 20.0) software was used to perform all the analyses.

## 3 RESULTS

### 3.1 The 3D reconstruction model

**FIGURE 1:**
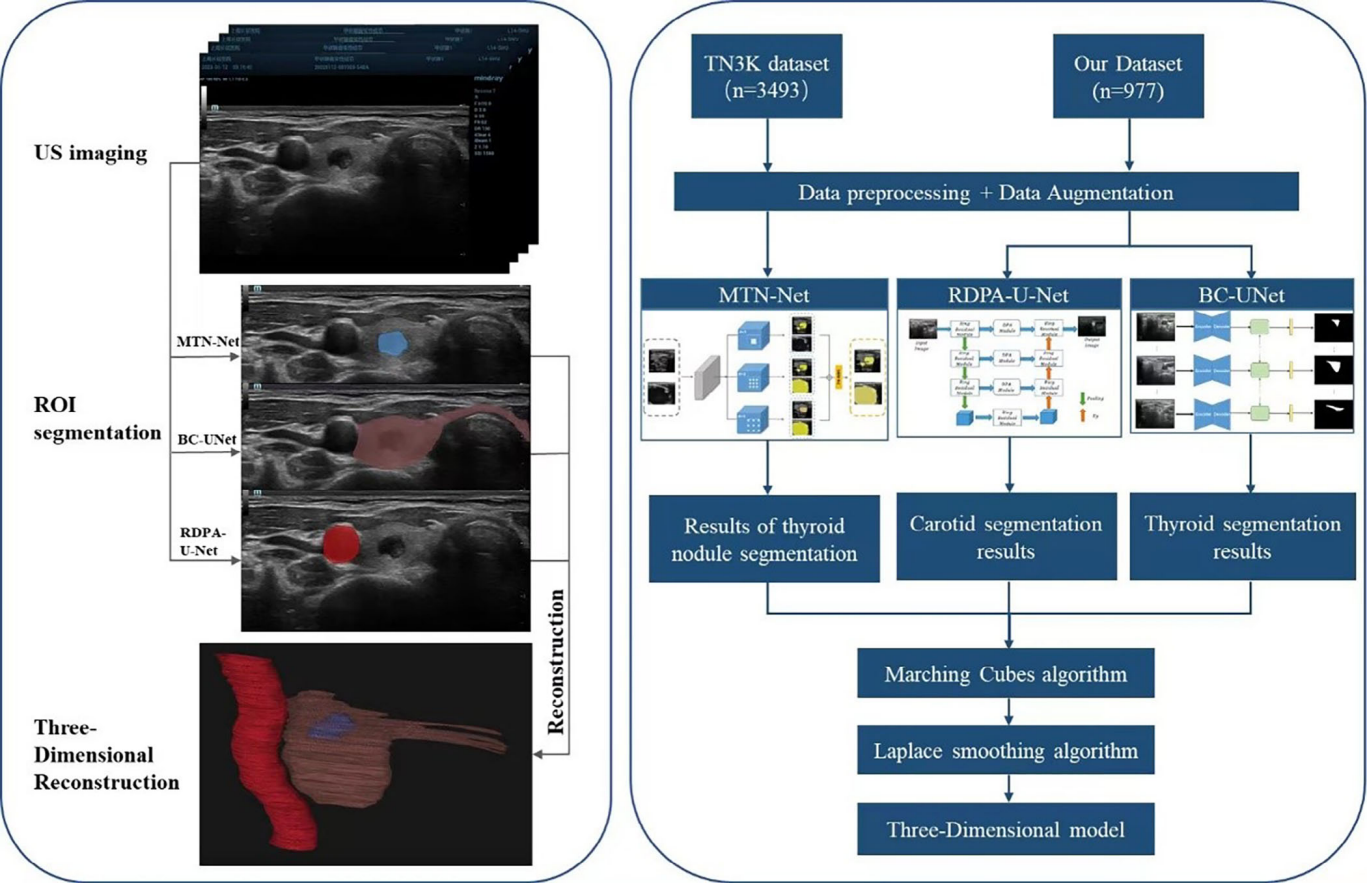
The 3D reconstruction workflow and algorithm flowchart. BC UNet, BiConvLSTM UNet; MTN Net, Multi‐Task Network; RDPA‐U‐Net, Ring Dual‐Path Attention U‐Net; ROI, region of interest.

**FIGURE 2:**
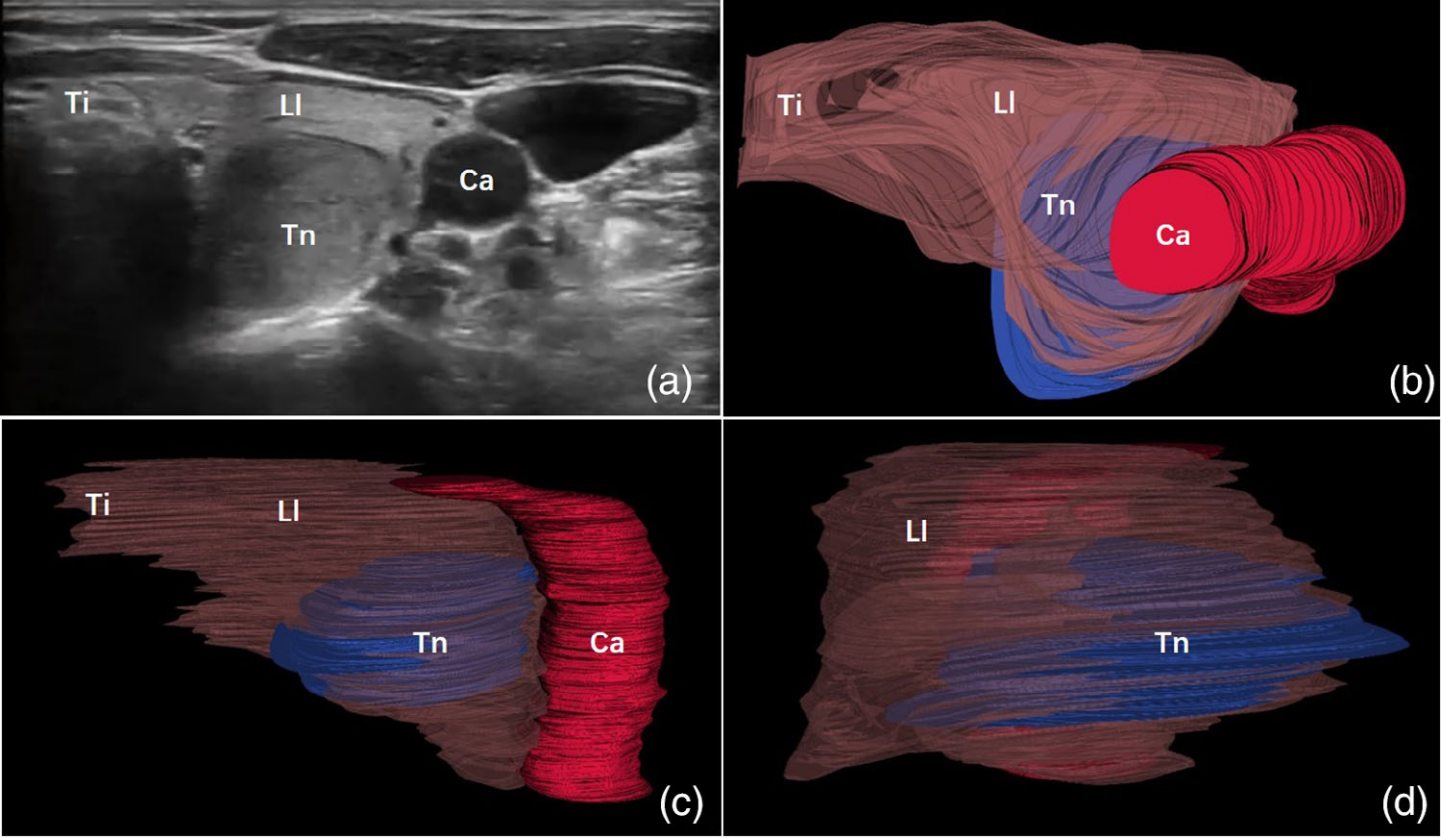
Images of a 34‐year‐old woman with a 38 mm thyroid nodule. The grayscale ultrasound image showed a thyroid nodule in the left thyroid lobe (a). The 3D reconstruction model could be rotated freely to get localization information of the nodule (b‐d). Images from the top view (b), front view (c), and side view (d) of the 3D reconstruction model were available. The nodule was shown in blue, the thyroid lobe was shown in brown and the carotid artery was shown in red. Ca, carotid artery; Ll, left lobe of thyroid gland; Ti, thyroid isthmus; Tn, thyroid nodule.

**FIGURE 3:**
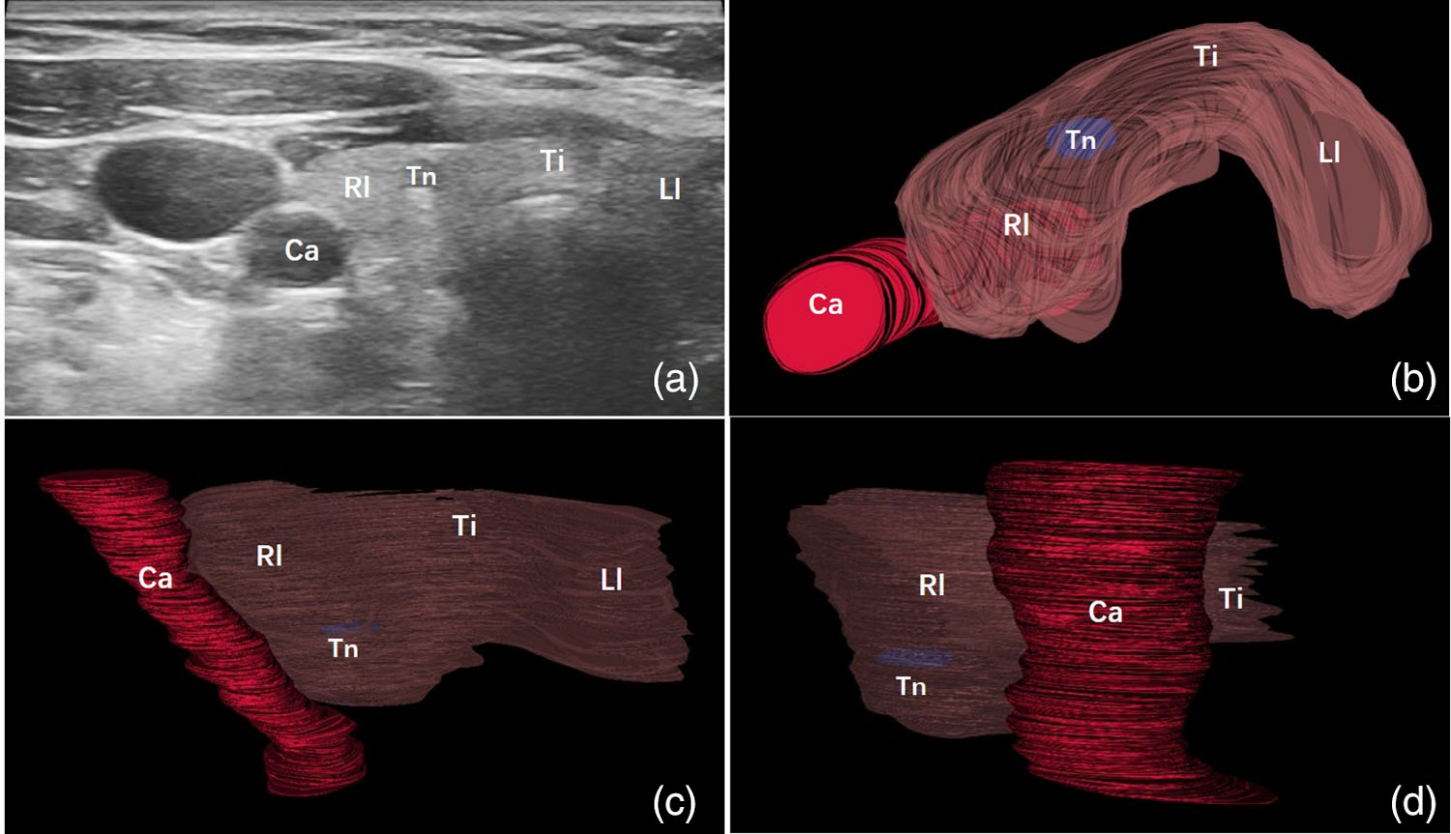
Images of a 26‐year‐old man with a thyroid nodule as small as 3 mm. The grayscale ultrasound image showed a thyroid nodule in the right thyroid lobe (a). The top view (b), front view (c), and side view (d) images from the 3D‐reconstructed model were available. Ca, carotid artery; Rl, right lobe of thyroid gland; Ti, thyroid isthmus; Tn, thyroid nodule.

**FIGURE 4:**
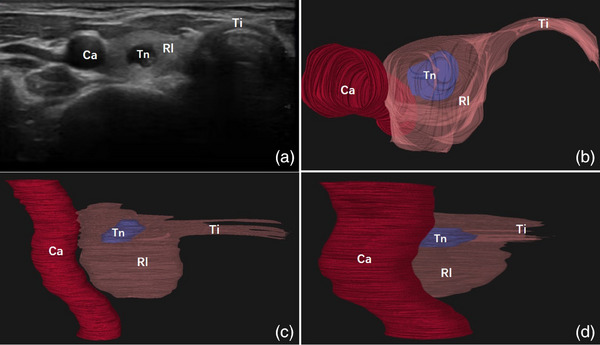
Images of a 43‐year‐old woman with a 9 mm cystic‐and‐solid thyroid nodule. The grayscale ultrasound image showed a thyroid nodule in the right thyroid lobe (a). The top view (b), front view (c), and side view (d) images from the 3D‐reconstructed model were available. Ca, carotid artery; Rl, left lobe of thyroid gland; Ti, thyroid isthmus; Tn, thyroid nodule.

Based on ultrasound videos, the unilateral thyroid gland, homolateral carotid artery, isthmus, and nodule were reconstructed and displayed with appropriate color and transparency. The 3D reconstruction workflow and algorithm flowchart are shown in Figure 1. The isthmus and carotid artery could help identify whether the thyroid gland was left or right. The shape, size, and location of the thyroid nodule as well as its relationship with thyroid capsule could be clearly displayed. Three examples of the 3D models generated from three patients implemented in the survey are shown in Figures 2, 3, 4. The reconstruction model could be rotated and the targeted nodules could be viewed from all angles. The patients and surgeons participated in this study could adjust the observation angle in real time according to their needs. A comprehensive understanding of the spatial location of nodule in the thyroid gland could be obtained. The nodules smaller than 5 mm could also be clearly identified (Figure 3).

### 3.2 The surveys

For the survey 1, five surgical residents, five attending surgeons, and five associate chief surgeons participated in the study. All of them were male. Table 1 summarizes the degree of surgeons understanding the spatial location of thyroid nodules before and after the 3D reconstruction model. Results showed that with the use of greyscale ultrasound images and report, there were differences in the understanding of the spatial location of thyroid nodules among the three groups of surgeons (*p = *0.027). The associate chief surgeons understood the anatomical location of thyroid nodules better than the surgical residents (*p < *0.05). Subsequently, all surgeons were provided with the corresponding 3D reconstruction model of the thyroid. The understanding of the thyroid gland anatomy considerably improved in all three groups (*p =* 0.007, 0.009, 0.005, respectively), with “fully understand” scores being attained. In particular, the degree of understanding the anatomical location of thyroid nodules did not differ among the three groups with the use of the 3D model (*p =* 0.619).

**TABLE 1:** Degree of understanding the spatial location of thyroid nodules without or with the 3D construction model in three separate groups.

	without 3D model (scores)	with 3D model (scores)	*p*
Surgical residents	1、2、3、3、2	5、5、5、4、5	0.007
Attending surgeons	2、3、3、4、3	5、5、4、5、5	0.009
Associate chief surgeons	3、4、3、4、4^*^	5、5、5、5、5	0.005
*p*	0.027	0.619	

* *p* < 0.05 vs. surgical residents.

For the survey 2, the clinicopathological parameters of patients taking the survey are summarized in Table 2. Ten patients were surveyed without the 3D reconstruction model (control group), while 10 patients were surveyed with the 3D reconstruction model (experimental group). The mean age was 45.62 ± 10.31 years for the control group and 47.23 ± 11.33 years for the experimental group (*p *= 0.643). The sex ratio in the two groups was comparable (*p* = 0.539). The mean tumor size was 8.40 ± 2.48 mm for the control group and 8.24 ± 3.57 mm for the experimental group (*p* = 0.875). Table 3 summarizes the degree of patients’ understanding of the spatial location of thyroid nodule and procedure for the thyroid surgery, as well as the time consumption of surgeon‐patient communication. The patients’ understanding of the location of thyroid nodule and procedure for thyroid surgery was significantly improved in the experimental group (*p* = 0.001), attaining a mean score of 4.55 ± 0.61 (indicating better than “understand”). In addition, with the use of 3D model, the time for surgeons explaining to patients was significantly reduced (16.75 ± 2.94  vs. 8.85 ± 1.66 min, *p* = 0.001).

**TABLE 2:** The clinicopathological parameters of patients participated in survey 2.

	without 3D model (*n* = 15)	with 3D model (*n* = 15)	*p*
Age (years)	45.62 ± 10.31	47.23 ± 11.33	0.643
Sex			0.539
Female	11	9	
Male	9	11	
Tumor size (mm)	8.40 ± 2.48	8.24 ± 3.57	0.875

**TABLE 3:** The degree of understanding related to the patient‐oriented survey and the communication time according to the explanation with or without the 3D model.

	without 3D model	with 3D model	*p*
The understanding of thyroid nodule location and the procedure for thyroid surgery (score)	2.05 ± 0.76	4.55 ± 0.61	0.001
Communication time (min)	16.75 ± 2.94	8.85 ± 1.66	0.001

## 4 DISCUSSION

A clear display of the spatial relationship of the thyroid nodules and the thyroid gland is important for surgical path planning and surgeon‐patient communication. However, the conventional thyroid ultrasound or 3D thyroid ultrasound failed to provide the spatial location of the nodular in the thyroid. To solve this problem, the investigators conducted studies involving 3D‐printed thyroid cancer phantom^4^ produced from segmented contrast‐enhanced CT, personalized 3D model produced from U‐Net‐based deep learning architecture based on data from CT,^6^ or spatial reconstruction of the thyroid based on ultrasound and CT imaging data.^13^ These attempts were limited for that CT was not a routine procedure for all thyroid surgeries. The potential radiation risk and cost should not be ignored. In our study, ultrasound videos, rather than CT, were innovatively employed for 3D model reconstruction since neck ultrasound was the routine preoperative imaging procedure for thyroid surgery. BC‐UNet, MTN‐Net, and RDPA‐U‐Net network models were innovatively employed for segmentation of the thyroid glands, the thyroid nodules, and the carotid artery respectively. Our work is a preliminary attempt of ultrasound 3D reconstruction imaging based on artificial intelligence. The thyroid glands, thyroid nodules, and carotid arteries could be automatically identified. To our knowledge, this is the first report of constructing a thyroid model using a deep learning technique for personalized thyroid segmentation based on ultrasound videos, with automatic detection of thyroid glands, thyroid nodules and carotid arteries.

In our study, the size and location of the thyroid nodule, as well as the anatomical relationship between the nodule and thyroid gland could be effectively presented in the personalized 3D reconstruction model. The reconstruction model had the ability to rotate the thyroid gland, thereby improving visualization of the thyroid nodule within the thyroid gland. The preliminary clinical application showed that it was conducive to the comprehension of the location of thyroid nodules for surgeons and patients, with significant improvement on the efficiency of surgeon‐patient communication.

The surgical procedures for thyroid tumors are relatively standardized (i.e., lobectomy or total thyroidectomy). In the real world, however, the extent of surgery is decided by a comprehensive assessment, including location (lobe or isthmus), tumor size (micro‐or macro‐), invasion into surrounding tissues, and patient preference.^14^ For some patients, they tend to operate directly without fine needle biopsy, or prefer conservative thyroidectomy, which involving partial removal of the thyroid cancer lesion rather than the entire ipsilateral thyroid lobe.^15^ In these cases, the surgeons need a better understanding of the anatomical location of the thyroid nodule to make surgical path planning, ensuring the proper scope of thyroidectomy during operation and avoiding nodules omission, especially for the small nodules.

Providing patients with sufficient information of their disease is crucial since patients’ preference play an important part in the surgical decision. However, most patients do not have enough medical knowledge. Surgeons had to explain the patient's condition by drawing images of the thyroid or through a plastic thyroid model, which proved rough and inaccurate. In 2021, Seok et al. used a U‐Net‐based deep learning architecture and a mesh‐type 3D modeling technique to fabricate the personalized 3D model based on data from CT.^6^ They found that the personalized 3D‐printed thyroid model was helpful to understand the disease, operation, and possible complications and their overall satisfaction. By using deep learning segmentation and a mesh‐type 3D modeling technique, the time and cost was reduced than traditional 3D‐printing procedure.^16^, ^17^ The work was limited for that CT might not be a routine procedure for all thyroid surgeries. The potential radiation risk should not be ignored.

In our study, ultrasound videos, rather than CT, was employed for 3D model reconstruction since neck ultrasound is the routine preoperative imaging procedure for thyroid surgery. For ultrasound video thyroid segmentation, we added ASPP module in BC‐UNet to obtain multiscale features of thyroid in ultrasound images, and used BiConvLSTM module to obtain forward and backward time series information of video. Finally, BC‐UNet trained, validated, and tested on our dataset, reaching 82.3% on dice coefficient. It exhibited better segmentation performance than the existing models (Table S1). Compared with 2D UNet^7^ and UNet++^18^ which only segmented single images without adding context information, the ASPP module added in BC‐UNet could effectively obtain multi‐scale information of the thyroid in ultrasound images, thereby accurately segmenting thyroids of different sizes. At the same time, compared with 3D UNet,^19^ although 3D UNet could obtain the correlation information between the upper and lower slices through 3D convolution operations, BC‐UNet obtained long‐distance information in the forward and backward time directions in the ultrasound cross‐sectional video through the BiConvLSTM module, thereby combining the features of multiple frames before and after to make the segmentation result of the current frame more accurate. For thyroid nodule segmentation of ultrasound images, we designed the thyroid nodule segmentation network MTN‐Net based on Trident Network, enabling the network to extract multi‐scale features of thyroid nodules and fuse the results of multiple branches by the TN‐NMS algorithm, which could effectively suppress false detection results. Finally, MTN‐Net was trained, validated, and tested on the publicly available dataset TN3K and achieved 56.8% on mAP@0.5:0.95. Compared with other models, MTN‐Net achieved great improvements in AP, AP50, APM, and APL. Compared with other models, MTN‐Net had achieved significant improvements in AP, AP50, APM, and APL (Table S2). However, in terms of the average accuracy of detecting and segmenting small thyroid nodules, MTN‐Net was inferior to Mask Scoring R‐ CNN^20^ and Point Rend.^21^ This was because the appearance and texture of some small thyroid nodules were very similar to the surrounding tissues, and MTN‐Net was prone to mis‐detect other tissues and organs as small thyroid nodules. However, MTN‐Net still had high accuracy in APM and APL, indicating that MTN‐Net had a significant advantage in detecting and segmenting medium and large thyroid nodules. In terms of carotid segmentation of ultrasound images, we used a ring residual module instead of a convolutional layer in the carotid segmentation network RDPA‐U‐Net and a dual‐path attention module to fuse multi‐scale spatial information, improving the segmentation accuracy of the carotid artery. Eventually RDPA‐U‐Net was trained, validated, and tested on our dataset, achieving Dice coefficient of 93.1%. It exhibited better performance than the existing models for the Dice coefficient of carotid artery segmentation (Table S3). In contrast to other models, RDPA‐U‐Net substituted the convolutional layer with a ring residual module, thereby effectively capturing intricate details within the image. Additionally, RDPA‐U‐Net incorporated a dual‐path attention module, which amalgamated multi‐scale spatial information, thereby enhancing its ability to handle complex structures and textures within the image, consequently augmenting the precision of carotid artery segmentation.

The accuracy of the model is of great significance for clinical work. For this reason, the thyroid segmentation model BC‐UNet and the thyroid nodule segmentation model MTN‐Net proposed in this paper were used to achieve accurate detection and segmentation of thyroid and its nodules. Through experimental verification, the Dice of thyroid segmentation reached 0.823, IoU reached 0.689, and the AP of thyroid nodule segmentation reached 56.8%, which was better than other excellent models. On this basis, the mature modeling methods MC (Marching Cubes) algorithm^12^ and Laplacian smoothing algorithm^22^ were used to achieve 3D modeling of thyroid and its nodules.

Compared with reading ultrasound reports and ultrasound images, the application of 3D models had improved the understanding of thyroid nodule's spatial location by surgeons at various professional levels, reaching a level of “understand” or above. Meanwhile, before using the 3D model, the associate chief surgeon group had a significantly better understanding of the spatial location of thyroid nodules than the surgical resident group. With the use of the 3D model, the impact was eliminated, and all surgeons with different professional levels achieved a high level of understanding. In addition, the patients’ understanding of the anatomical location of thyroid nodule and procedure for thyroid surgery was significantly improved in the group with the 3D model. In particular, with the use of 3D reconstruction model, the time for surgeons to explain to patients was significantly reduced.

For the diagnosis and treatment of thyroid nodules, ultrasound is the indispensable imaging method. However, due to the specialty of ultrasound, the information provided by ultrasound, especially the spatial information, could not be fully attained by the clinicians. Nowadays, with the development of artificial intelligence technology, it is hopeful to shorten the distance between ultrasound and clinicians and provide more ultrasound information for clinicians. This study is a preliminary attempt of ultrasound 3D reconstruction imaging based on artificial intelligence. In the future research, a 3D reconstruction of bilateral thyroid, bilateral thyroid nodules, and surrounding soft tissues would bring more help for the clinicians and patients.

There is still some limitation of our study. Considering ultrasound image quality and clarity required, the ultrasound video used for model construction only record unilateral thyroid, isthmus, and ipsilateral carotid artery. The contralateral thyroid and the contralateral carotid artery were not included. Therefore, for patients with bilateral nodules, two reconstruction models were required.

## 5 CONCLUSION

We established the methodology of personalized 3D reconstruction thyroid model using multi‐Net based on deep learning approach and the marching cubes algorithm techniques based on ultrasound videos, which noninvasively provide a visual representation of the spatial relationship of the thyroid nodules and the thyroid gland. We confirmed the usefulness of this model on improving the understanding of the anatomical location of thyroid nodules for surgeons and patients. In addition, the 3D reconstruction model could improve patients’ understanding of the disease and surgery, with significant improvement on the efficiency of surgeon‐patient communication.

## AUTHOR CONTRIBUTIONS

Guarantor of integrity of the entire study: Huang Hejing, Hu Wenxin; study concepts and design: Huang Hejing; literature research: Huang Yandong, Zhang Shiqi; data collection: Huang Yandong, Jia Lanting; model construction: Hu Wenxin, Chen Leyao; data analysis: Huang Yandong; manuscript preparation: Huang Yandong, Huang Hejing; manuscript review: Huang Hejing. All authors read and approved the final manuscript.

## CONFLICT OF INTEREST STATEMENT

The authors declare no conflict of interest.

## Supporting information

Supplementary Figure 1. The model structure diagram of BC‐UNet.

Supplementary Figure 2. The model structure diagram of MTN‐Net.

Supplementary Figure 3. The model structure diagram of RDPA‐U‐Net.

Supporting Information

Supporting Information

Supporting Information
