# Supplementary figures and images for: Establishment and preliminary application of personalized three‐dimensional reconstruction of thyroid gland with automatic detection of thyroid nodules based on ultrasound videos
Source: J Appl Clin Med Phys. 2024 Mar 25;25(6):e14332. doi: 10.1002/acm2.14332 (PMC11163481; doi:10.1002/acm2.14332)

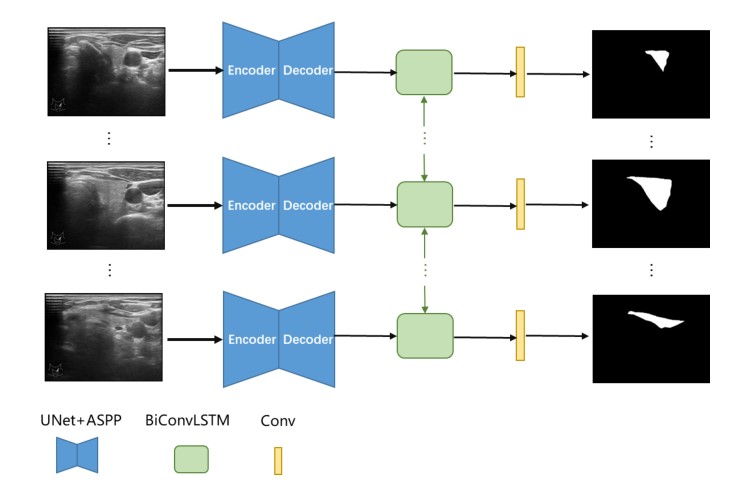

Supplement: Supplementary file 1 — Supplementary Figure 1. The model structure diagram of BC‐UNet. [file ACM2-25-e14332-s004.jpg]

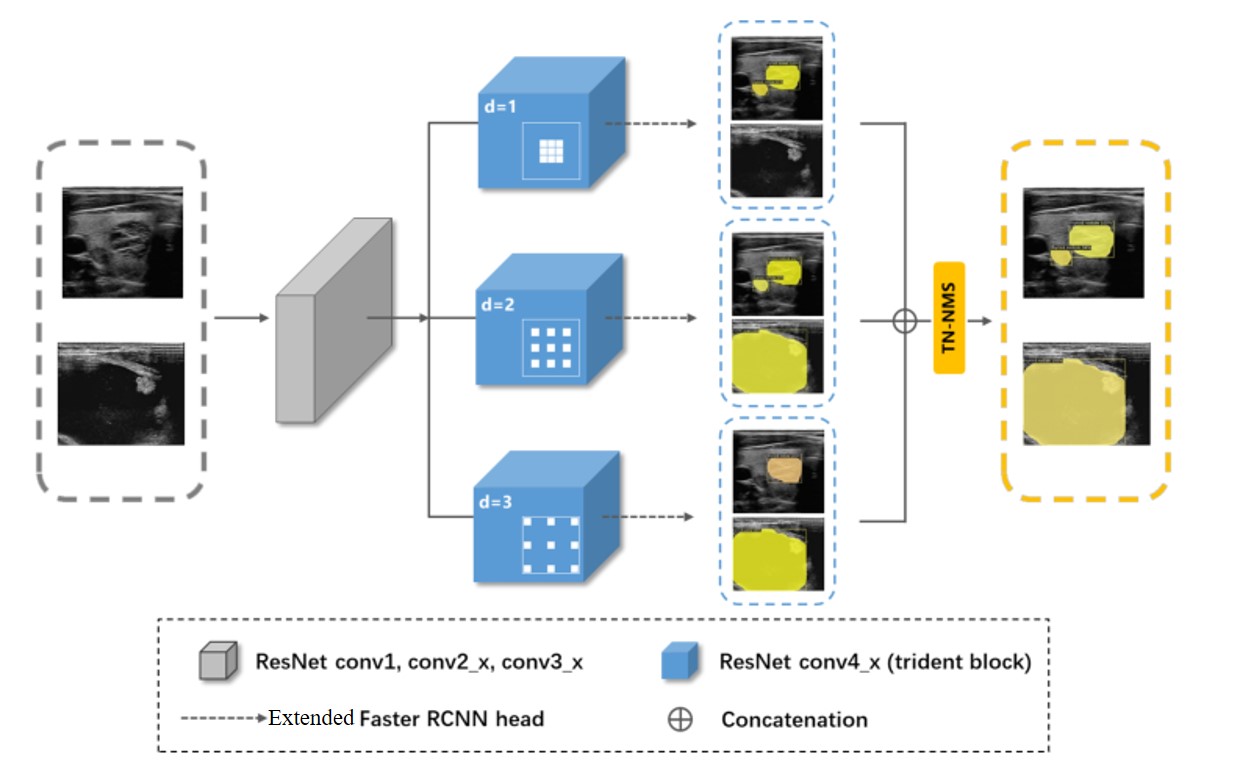

Supplement: Supplementary file 2 — Supplementary Figure 2. The model structure diagram of MTN‐Net. [file ACM2-25-e14332-s005.jpg]

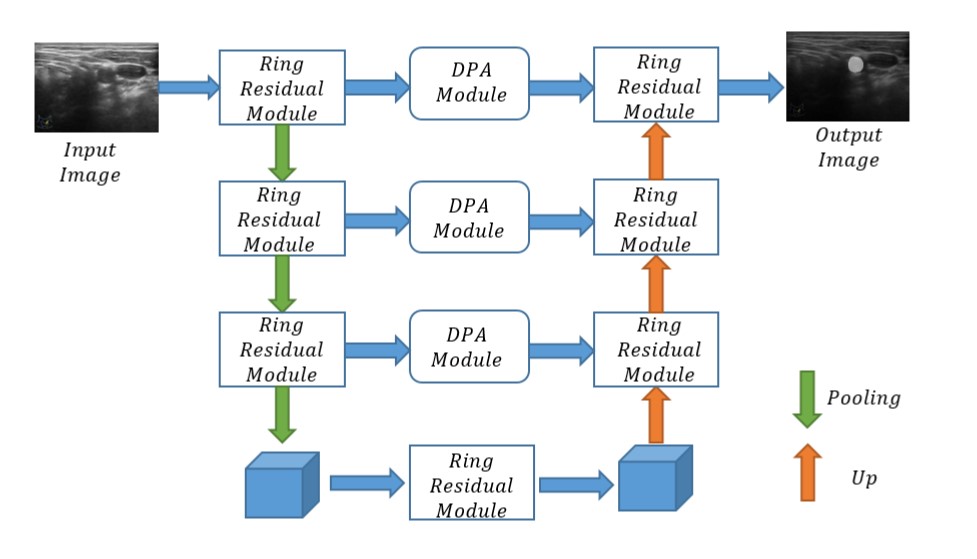

Supplement: Supplementary file 3 — Supplementary Figure 3. The model structure diagram of RDPA‐U‐Net. [file ACM2-25-e14332-s002.jpg]
