# Supplementary material for: Establishment and preliminary application of personalized three‐dimensional reconstruction of thyroid gland with automatic detection of thyroid nodules based on ultrasound videos
Source: J Appl Clin Med Phys. 2024 Mar 25;25(6):e14332. doi: 10.1002/acm2.14332 (PMC11163481; doi:10.1002/acm2.14332)
Supplement: Supplementary file 4 — Supporting Information [file ACM2-25-e14332-s003.docx]

**Supplementary Table 1 Comparison of the performance of models in thyroid segmentation experiment.**

| Model | Dice | IoU |
| --- | --- | --- |
| 2D U-Net | 0.770 | 0.637 |
| 3D U-Net | 0.721 | 0.594 |
| U-Net++ | 0.799 | 0.668 |
| **BC U-Net** | **0.823** | **0.689** |
