# Supplementary material for: Establishment and preliminary application of personalized three‐dimensional reconstruction of thyroid gland with automatic detection of thyroid nodules based on ultrasound videos
Source: J Appl Clin Med Phys. 2024 Mar 25;25(6):e14332. doi: 10.1002/acm2.14332 (PMC11163481; doi:10.1002/acm2.14332)
Supplement: Supplementary file 5 — Supporting Information [file ACM2-25-e14332-s001.docx]

**Supplementary Table 2. Comparison of the results of different methods in thyroid nodule segmentation.**

| Model | TN3K | | | | | DDTI | | | |
| --- | --- | --- | --- | --- | --- | --- | --- | --- | --- |
|  | AP | AP_50_ | AP_S_ | AP_M_ | AP_L_ | AP | AP_50_ | AP_M_ | AP_L_ |
| Mask R-CNN | 54.4 | 84.5 | 30.0 | 48.3 | 60.7 | 42.6 | 80.2 | 36.4 | 49.7 |
| Cascade Mask R-CNN | 55.5 | 84.7 | 31.6 | 49.8 | 62.0 | 45.6 | 84.4 | 41.0 | 51.0 |
| Mask Scoring R-CNN | 55.1 | 84.6 | 37.5 | 49.5 | 61.1 | 46.1 | 82.9 | 41.5 | 51.9 |
| PointRend | 56.2 | 85.5 | 36.5 | 50.3 | 62.3 | 46.4 | 81.0 | 39.5 | 53.2 |
| **MTN-Net** | **56.8** | **86.9** | 35.5 | **50.8** | **62.9** | **49.0** | **86.6** | **44.7** | **54.4** |
