# Supplementary material for: Establishment and preliminary application of personalized three‐dimensional reconstruction of thyroid gland with automatic detection of thyroid nodules based on ultrasound videos
Source: J Appl Clin Med Phys. 2024 Mar 25;25(6):e14332. doi: 10.1002/acm2.14332 (PMC11163481; doi:10.1002/acm2.14332)
Supplement: Supplementary file 6 — Supporting Information [file ACM2-25-e14332-s006.docx]

**Supplementary Table 3. Comparison of the results of different methods in cervical tubular organ segmentation**

| Model | Carotid Artery Dice | Jugular Vein Dice | Trachea Dice |
| --- | --- | --- | --- |
| U-Net | 0.862 | 0.875 | 0.824 |
| U-Net++ | 0.890 | 0.892 | 0.862 |
| Attention U-Net | 0.876 | 0.889 | 0.857 |
| **RDPA-U-Net** | **0.931** | **0.912** | **0.891** |
